# Haploidentical transplantation combined with mesenchymal stem cells co-infusion improves survival in severe aplastic anemia: a single-center retrospective study

**DOI:** 10.3389/fmed.2025.1711958

**Published:** 2025-11-28

**Authors:** Zhengwei Tan, Miaoya Le, Ningning Zhu, Jingjing Liu, Yuechao Zhao, Huijin Hu, Qinghong Yu, Yu Zhang, Liqiang Wu, Tonglin Hu, Dijiong Wu, Baodong Ye, Wenbin Liu

**Affiliations:** 1 Department of Hematology, The First Affiliated Hospital of Zhejiang Chinese Medical University (Zhejiang Provincial Hospital of Traditional Chinese Medicine), Hangzhou, China; 2 The First School of Clinical Medicine, Zhejiang Chinese Medical University, Hangzhou, China

**Keywords:** aplastic anemia, haploidentical hematopoietic stem cell transplantation, mesenchymal stem cells, graft-versus-host disease, overall survival

## Abstract

**Background:**

Haploidentical hematopoietic stem cell transplantation (HID-HSCT) serves as an alternative treatment for severe aplastic anemia (SAA) patients lacking a suitable HLA-identical sibling donor. Compared to HLA-matched HSCT, HID-HSCT has higher rates of graft failure (GF) and graft-versus-host disease (GVHD). Recent studies suggest promising clinical outcomes when Mesenchymal stem cells (MSCs) are combined with HID-HSCT for SAA treatment.

**Methods:**

This study retrospectively analyzed clinical data from 190 SAA patients who underwent HID-HSCT with or without MSCs co-infusion. Patients were divided into two groups: the HID group (100 patients receiving only HID-HSCT) and the HID+MSC group (90 patients receiving HID-HSCT combined with MSC co-infusion).

**Results:**

The analysis revealed that the HID+MSC group had a significantly higher 5-year overall survival rate compared to the HID group (86.6% vs. 75.0%, *p* = 0.036) and a significantly improved GRFS rate (76.6% vs. 64.0%, *p* = 0.048). While MSCs co-infusion did not significantly reduce the incidence of aGVHD or cGVHD, a downward trend was observed, particularly for cGVHD (16.6% vs. 26.0%). Both groups showed high cumulative engraftment rates for NE and PLT within 28 days post-transplant, with no significant differences. Regarding viral reactivation, EBV and CMV reactivation rates were similar between the two groups, though four patients in the HID group developed EBV-associated PTLD.

**Conclusion:**

This study demonstrates that combining HID-HSCT with MSCs co-infusion is a safe and effective therapeutic strategy that significantly improves survival rates and quality of life in SAA patients.

## Introduction

Aplastic anemia (AA) is an acquired bone marrow failure disorder characterized primarily by pancytopenia, resulting from the immune-mediated destruction of hematopoietic stem and progenitor cells. For young AA patients who have an HLA-matched sibling donor, allogeneic hematopoietic stem cell transplantation (allo-HSCT) is the preferred treatment option (1–3). However, many patients lack HLA-matched sibling donors (MSD). In such cases, matched unrelated donors (MUD) or haploidentical donors (HID) have emerged as viable alternative options for transplantation (4–6). Over the past decade, granulocyte colony-stimulating factor (G-CSF) and antithymocyte globulin (ATG)-based HID-HSCT has achieved survival outcomes comparable to those observed in MSD-HSCT. However, graft failure (GF) and graft-versus-host disease (GVHD) are two critical factors that significantly impact the efficacy of HID-HSCT and long-term survival in AA patients (7).

Mesenchymal stem cells (MSCs) are a type of multipotent non-hematopoietic progenitor cells capable of interacting with hematopoietic stem cells (HSCs) and providing essential stromal support and growth factors for their proliferation. Furthermore, MSCs exhibit immunomodulatory functions that can inhibit immune rejection, promote immune reconstitution, and reduce the risk of GVHD (8–12). MSCs co-infusion as an adjunctive therapy has become an important strategy to improve transplant outcomes in AA patients. Due to HLA haploidentity, HID-HSCT is often associated with issues such as graft failure and post-transplant infections. The MSCs co-infusion can support the proliferation and differentiation of hematopoietic stem cells by secreting various cytokines (e.g., IL-6, VEGF), thereby optimizing the transplant microenvironment and accelerating hematopoietic recovery. Meanwhile, MSCs can increase the proportion of regulatory T cells (Tregs), induce immune tolerance, reduce the incidence of GVHD, and contribute to thymic repair, thereby enhancing homeostatic regulation of the immune system. Multiple clinical studies have confirmed that MSCs significantly improve stem cell engraftment and long-term survival in aplastic anemia patients undergoing haploidentical transplantation (13, 14). This study retrospectively analyzed the clinical data of 190 AA patients who underwent HID-HSCT combined with MSCs at our center in recent years, aiming to systematically evaluate the efficacy and safety of MSC co-infusion.

## Patients and methods

### Patients

The present study is a single-center, retrospective clinical analysis. It encompassed a review of the clinical data from 190 patients with a diagnosis of AA. These patients underwent HID-HSCT at the Hematology Department of The First Affiliated Hospital of Zhejiang Chinese Medical University between Jan 2018 and May 2025. The patients were categorized into two groups: 90 patients received MSCs co-infusion and 100 patients received only HID-HSCT. The study was reviewed and approved by the Ethics Committee of The First Affiliated Hospital of Zhejiang Chinese Medical University (Approval number: 2024-KLs-593-01), and all patients and donors provided written informed consent prior to treatment. The study adhered to the guidelines of the Declaration of Helsinki.

The inclusion criteria were as follows: (1) confirmed diagnosis of AA; (2) Treatment period between Jan 2018 and May 2025; (3) underwent HID-HSCT; (4) availability of complete clinical data.

### Conditioning regimen

The conditioning regimen involved in this study consist of the Bucy regimen, the FCA regimen, and the novel FABT regimen developed by our center. Details are as follows: Bucy regimen: −7d to −6d, Busulfan 3.2 mg/kg/d; −5d to −2d, cyclophosphamide (CTX) 50 mg/kg/d; −5d to −2d, ATG 2.5 mg/kg/d. FCA regimen: −9d to −5d, Fludarabine 30 mg/m^2^/d; −5d to −2d, CTX 50 mg/kg/d; −5d to −2d, ATG 2.5–3.5 mg/kg/d. FABT regimen: −7d to −5d, ATG 2 mg/kg/d; −7d to −3d, Fludarabine 30 mg/m^2^/d; −4d, Busulfan 0.8 mg/kg/d; −3d to −2d, CTX 25 mg/kg/d; −2d, Thiotepa 5 mg/kg.

### GVHD prophylaxis

GVHD prophylaxis in the FABT group included high-dose CTX, cyclosporine A (CsA) or tacrolimus (FK506), and mycophenolate mofetil (MMF). The specific regimen was as follows: +3d to +4d, CTX 40 mg/kg/d. In the FCA group and the Bucy group, GVHD prophylaxis was based on a short course of methotrexate (MTX), CsA (or FK506), and MMF. The specific regimen was as follows: +1d, MTX 15 mg/m^2^; +3d, +6d, +11d, MTX 10 mg/m^2^.

### Stem cell source and infusion protocol

The graft source mainly comprised G-CSF-mobilized Peripheral Blood Stem Cells (PBSCs) and/or bone marrow stem cells (BMSCs). Bone marrow-mesenchymal stem cells (BM-MSCs) were provided by Shandong Provincial Cord Blood Bank at a dose of 1 × 10^6^/kg. We routinely recommend MSCs for all patients to improve transplant tolerance. However, some patients still decline MSC treatment due to factors such as financial constraints. On day −1, MSCs were infused 6 h prior to donor stem cell transfusion and used only once. BMSCs were administered on day 01, followed by PBSCs on day 02. All procedures were performed in accordance with standardized clinical protocols for stem cell mobilization, collection, and infusion.

### End points and definitions

All outcomes were defined from the time of the first HSCT. The primary endpoints were GVHD-free and relapse-free survival (GRFS) and overall survival (OS). Secondary endpoints included engraftment, incidence of GVHD, and viral reactivation.

Neutrophil (NE) Engraftment: By day 28 post-transplantation, an absolute neutrophil count reaching 0.5 × 10^9^/L on three consecutive days. Platelet (PLT) Engraftment: By day 28 post-transplantation, a platelet count maintained above 20 × 10^9^/L for seven consecutive days without transfusion support. Acute GVHD (aGVHD) and Chronic GVHD (cGVHD): Classified and graded according to the internationally recognized Glucksberg-Seattle criteria (15). CMV Reactivation: CMV Reactivation: CMV-DNA copy number in plasma sample exceeding 1,000 copies/mL on two separate occasions. EBV Reactivation: EBV-DNA copy number in whole blood samples exceeding 10,000 copies/mL on two consecutive occasions. GRFS: survival without disease relapse or grade III-IV aGVHD or extensive cGVHD. OS: the time from HSCT to death or last follow-up.

### Statistical analysis

Data analysis was performed using SPSS version 27.0 (IBM, Armonk, NY) and Prism version 8.0 (GraphPad Software, San Diego, CA). Continuous variables with non-normal distributions were presented as the median (range) and analyzed using the Mann–Whitney U test (rank-sum test). Categorical variables were expressed as frequencies and analyzed using the chi-square test. Survival analysis was conducted using the Kaplan–Meier method, with the log-rank test used to compare survival curves. Univariate and multivariate Cox regression analyses were applied to evaluate the prognostic risk factors for survival data. Factors with a *p*-value <0.1 in the univariate analysis, along with those previously identified in the literature as predictors of poor GRFS outcomes, were incorporated into a multivariate Cox regression model. Statistical significance was set at a *p*-value < 0.05.

## Results

### Patient characteristics and transplantation outcomes

Patients were divided into two groups: HID group (100 patients) and HID+MSC group (90 patients), according to whether MSCs co-infusion was administered. No statistically significant differences were observed among the groups in terms of demographic and clinical characteristics, including gender ratio, age distribution, diagnosis, blood type, conditioning regimen, and graft source (*p* > 0.05). For further details, please refer to Table 1.

**Table 1:** Baseline data of two groups.

Characteristic	HID group	HID+MSC group	*p*
Number	100	90	
Sex, *n* (%)			0.493
Male	55 (55)	45 (50)	
Female	45 (45)	45 (50)	
Age, yr., median (range)	31 (8 ~ 74)	31 (8 ~ 66)	0.924
Donor sex, *n* (%)			0.837
Male	63 (63)	58 (64.5)	
Female	37 (37)	32 (35.5)	
Donor, Age, yr., median (range)	28 (5 ~ 55)	28 (7 ~ 54)	0.893
Blood values before HSCT, median (range)			
WBC	1.7 (0.1 ~ 3.4)	1.7 (0 ~ 3.4)	0.193
ANC	0.4 (0 ~ 1.4)	0.4 (0 ~ 2.1)	0.205
HGB	57 (25 ~ 74)	57 (14 ~ 77)	0.518
PLT	12 (1 ~ 38)	11.5 (1 ~ 44)	0.171
Ferritin	792 (37 ~ 17,747)	792 (52 ~ 12,374)	0.144
Disease classification, *n* (%)			0.278
SAA	51 (55.4)	53 (58.9)	
VSAA	50 (44.6)	37 (41.1)	
Interval from diagnosis to HSCT, *n* (%)			0.659
≤1 yr	51 (51)	63 (70)	
>1 yr	49 (49)	27 (30)	
HCT-CI, *n* (%)			0.644
0	68 (68)	64 (71.1)	
≥1	32 (32)	26 (28.9)	
DSA, *n* (%)			0.367
Negative	83 (83)	70 (77.8)	
Positive	17 (17)	20 (22.2)	
ABO mismatch, *n* (%)			0.951
Match	56 (56)	50 (55.6)	
Mismatch	44 (44)	40 (44.4)	
PNH, *n* (%)	7 (7)	8 (8.9)	0.632
IST, *n* (%)	3 (3)	5 (5.6)	0.384
HLA, *n* (%)			0.312
5/12	14 (14)	20 (22.2)	
6/12	45 (45)	38 (42.2)	
7/12	27 (27)	24 (26.7)	
8/12	9 (9)	5 (5.6)	
9/12	5 (5)	3 (3.3)	
Donor/Recipient EBV serology, *n* (%)			0.639
+/+	100 (100)	87 (96.7)	
−/+	NA	3 (3.3)	
Donor / Recipient CMV serology, *n* (%)			0.542
+/+	99 (99)	90 (100)	
−/+	1 (1)	NA	
Graft source, *n* (%)			0.216
PBSCs	9 (9)	4 (4.4)	
BMSCs + PBSCs	91 (91)	86 (95.6)	
Conditioning regimen, *n* (%)			0.089
FABT	25 (25)	20 (22.2)	
FCA	69 (69)	55 (61.1)	
Bucy	6 (6)	15 (16.7)	
CD34^+^ cell count, ×10^6^/kg, median (range)	6.0 (2.11 ~ 19.21)	6.0 (1.69 ~ 25.38)	0.587
Letermovir, *n* (%)	24 (24)	21 (23.3)	0.915

Significant *p* values are in bold type.

WBC, white blood cell; ANC, absolute neutrophil count; HGB, hemoglobin; PLT, platelet; PBSCs, peripheral blood stem cell; BMSCs, bone marrow stem cell; DSA, donor specific antibody; PNH, paroxysmal nocturnal hemoglobinuria; IST, immunosuppressive therapy; HCT-CI, hematopoietic cell transplantation–specific comorbidity index; HLA, human leukocyte antigen.

### Engraftment

The median number of CD34 + cells in the grafts was 6.0 × 10^6^/kg (range: 2.11 ~ 19.21) for the HID group and 6.0 × 10^6^/kg (range: 1.69 ~ 25.38) for the HID+MSC group, with no significant difference between the two groups (*p* = 0.587).

**Figure 1:**
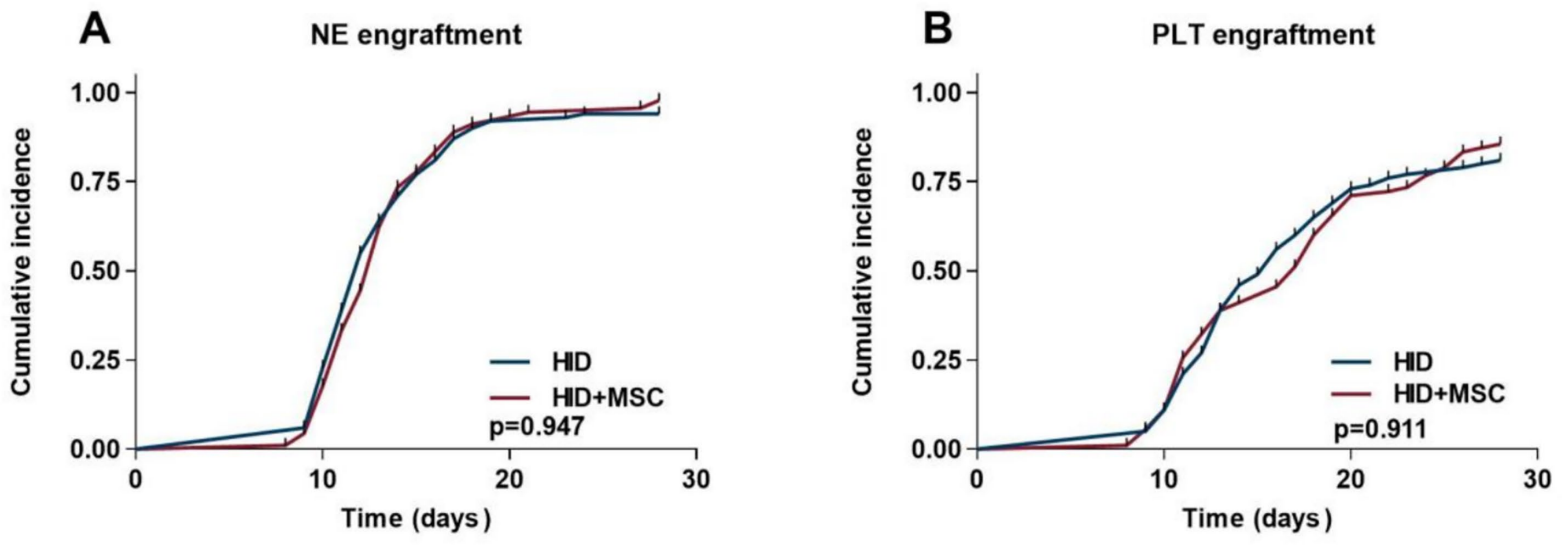
Transplantation outcomes after HID-HSCT in two groups. **(A)** NE engraftment. **(B)** PLT engraftment.

The cumulative NE engraftment rate within 28 days was 94.0% (95%CI, 89.1% ~ 99.6%) in the HID group and 97.7% (95% CI, 95.1% ~ 100%) in the HID+MSC group (*p* = 0.947) (Figure 1A). The cumulative PLT engraftment rate within 28 days was 81.0% (95% CI, 73.1% ~ 89.2%) in the HID group and 85.5% (95%CI, 78.8% ~ 93.7%) in the HID+MSC group (*p* = 0.911) (Figure 1B).

### GVHD

**Figure 2:**
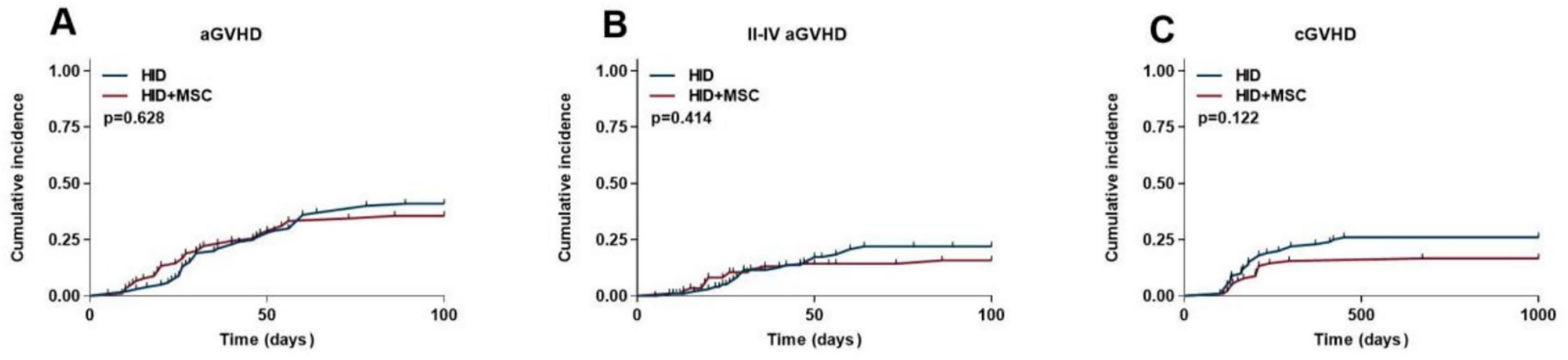
Transplantation outcomes after HID-HSCT in two groups. **(A)** aGVHD. **(B)** II-IV aGVHD. **(C)** cGVHD.

The cumulative aGVHD rate was 41.0% (95% CI, 31.1% ~ 51.6%) in the HID group and 35.5% (95% CI, 25.5% ~ 46.7%) in the HID+MSC group (*p* = 0.628) (Figure 2A). The cumulative II-IV aGVHD rate was 20.0% (95% CI, 12.1% ~ 28.1%) in the HID group and 14.4% (95% CI, 7.5% ~ 22.1%) in the HID+MSC group (*p* = 0.414) (Figure 2B). The cumulative cGVHD rate was 26.0% (95% CI, 17.1% ~ 35.9%) in the HID group and 16.6% (95% CI, 9.1% ~ 24.5%) in the HID+MSC group (*p* = 0.122) (Figure 2C).

### Virus reactivation

**Figure 3:**
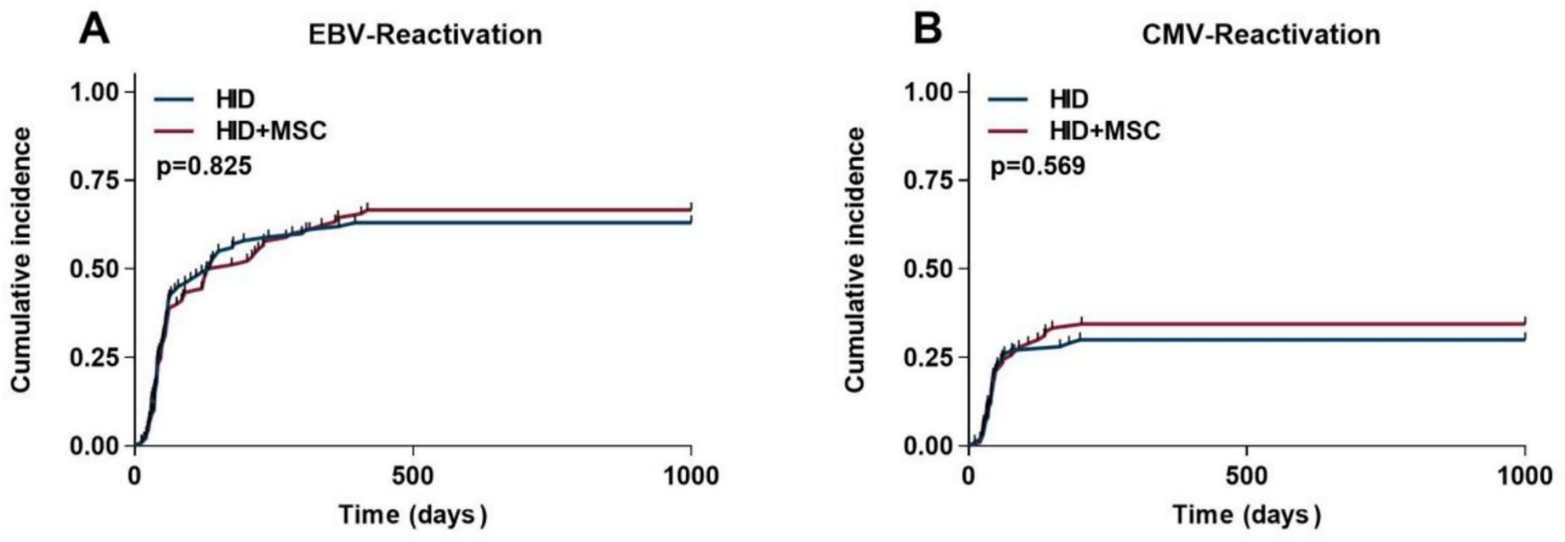
Transplantation outcomes after HID-HSCT in two groups. **(A)** EBV-reactivation. **(B)** CMV-reactivation.

The cumulative EBV reactivation rate was 63.0% (95% CI, 53.1% ~ 73.6%) in the HID group and 66.6% (95% CI, 57.5% ~ 77.7%) in the HID+MSC group (*p* = 0.825) (Figure 3A). Notably, despite the comparable rates of EBV reactivation between the two groups, 4 patients in the HID group developed EBV-associated post-transplant lymphoproliferative disorders (PTLD) (*p* = 0.045). Similarly, the cumulative CMV reactivation rate was 30.0% (95% CI, 21.7% ~ 39.9%) in the HID group and 34.4% (95% CI, 24.5% ~ 44.1%) in the HID+MSC group (*p* = 0.569) (Figure 3B). However, no patients in either group progressed to CMV disease.

### Efficacy and survival

**Figure 4:**
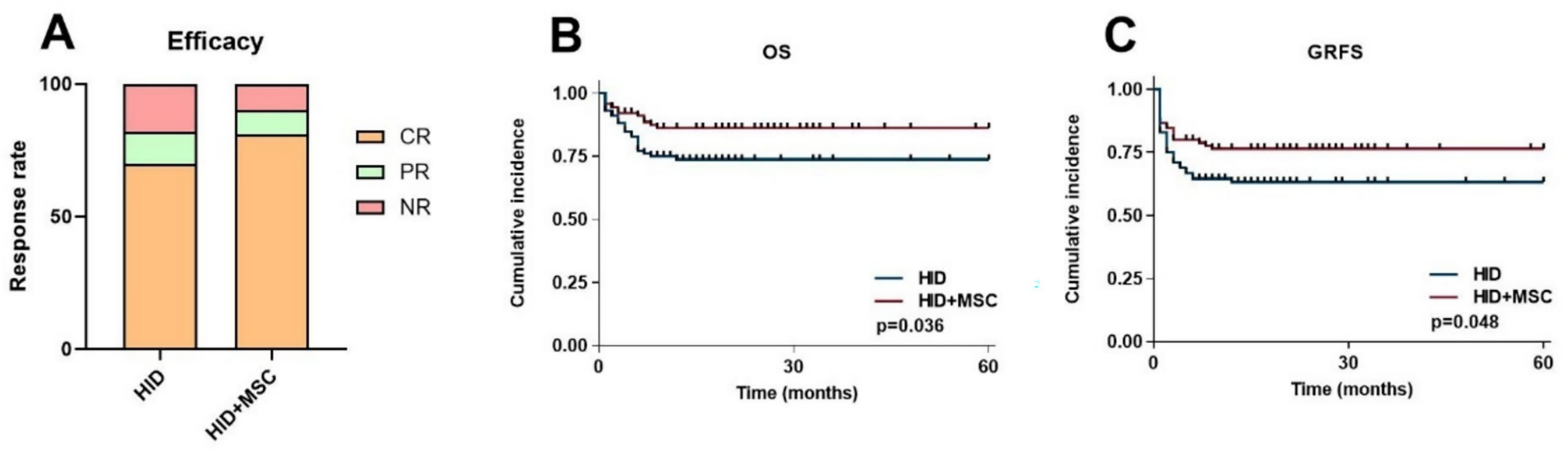
Transplantation outcomes after HID-HSCT in two groups. **(A)** Efficacy. **(B)** OS. **(C)** GRFS.

One year post-transplantation, the HID and HID+MSC groups had OR rates of 82.0% (95% CI, 71.6% ~ 92.7%) and 90.0% (95% CI, 86.7% ~ 99.6%) respectively, with no statistically significant difference (*p* = 0.120) (Figure 4A). The 5-year OS was 75.0% (95% CI, 66.6% ~ 84.7%) in the HID group compared to 86.6% (95% CI, 80.6% ~ 94.1%) in the HID+MSC group (*p* = 0.036) (Figure 4B). The 5-year GRFS was 64.0% (95% CI, 54.6% ~ 74.1%) in the HID group compared to 76.6% (95% CI, 68.1% ~ 86.3%) in the HID group (*p* = 0.048) (Figure 4C).

### Post-transplantation complications

We supplemented Table 2 with data on post-transplantation complications such as hemorrhagic cystitis and graft failure, showing that the HID+MSC group had relatively lower rates in these complications compared to the HID group.

**Table 2:** The incidence of Post-transplantation adverse events in two groups.

Complications	HID group	HID+MSC group	p
N	100	90	
HC	17 (17)	13 (14.4)	0.632
PGF	6 (6)	2 (2.2)	0.187
SGF	6 (6)	3 (3.3)	0.206
PT	19 (19)	13 (14.4)	0.405
TMA	2 (2)	1 (1.1)	0.626
SOS	1 (1)	0 (0)	0.344

HC, hemorrhagic cystitis, PGF, primary engraftment failure, SGF, secondary engraf failure, PT, persistent thrombocytopenia, TMA, thrombotic microangiopathy, SOS, hepatic sinusoidal obstruction syndrome.

### Univariate and multivariate analysis

In the univariate analysis of GRFS, we included factors such as patient age and sex, donor age and sex, diagnosis, MSC co-infusion, and HCT-CI. The results demonstrated that the absence of MSC co-infusion, HCT-CI ≥ 1, CMV reactivation, poor NE and PLT engraftment, Bucy and FCA regimen were independent risk factors for GRFS. Factors with a *p*-value <0.1 in the univariate analysis, along with those previously identified in the literature as predictors of poor GRFS outcomes, were incorporated into a multivariate Cox regression model. The analysis revealed that PLT engraftment [HR = 0.34, 95% CI (0.17–0.71), *p* = 0.004] and the FABT regimen [HR = 0.41, 95% CI (0.18–0.97), *p* = 0.044] were identified as protective factors for GRFS. For further details, please refer to Table 3.

**Table 3:** Univariate and multivariate analysis of the factors associated with GRFS.

Variable	Univariate analysis		Multivariate analysis	
	HR (95% CI)	*p* value	HR (95% CI)	*p* value
Patient sex, Male vs. Female	1.43 (0.84–2.44)	0.178	1.01 (0.56–1.79)	0.978
Patient age, ≤40 yr. vs. >40 yr	1.33 (0.79–2.24)	0.282	0.88 (0.46–1.68)	0.714
Donor sex, Male vs. Female	1.01 (0.59–1.73)	0.958	0.73 (0.38–1.37)	0.331
Donor age, ≤40 yr. vs. >40 yr	0.79 (0.41–1.51)	0.474	0.90 (0.42–1.89)	0.786
Diagnosis, SAA vs. VSAA	1.26 (0.75–2.11)	0.384	1.26 (0.69–2.31)	0.443
Time from diagnosis to HSCT, ≤1 yr. vs. >1 yr	1.08 (0.62–1.87)	0.784	1.46 (0.78–2.74)	0.235
Previous ATG treatment, No vs. Yes	0.38 (0.05–2.73)	0.355	0.42 (0.05–3.33)	0.410
MSC, Yes vs. No	0.60 (0.35–1.03)	**0.065**	1.10 (0.58–2.08)	0.759
HCT-CI, ≥1 vs. 0	1.66 (0.98–2.82)	**0.060**	0.96 (0.49–1.86)	0.913
Stem Cell Source, PB + BM vs. PB	0.98 (0.35–2.71)	0.970	0.76 (0.26–2.23)	0.621
ABO, Match vs. Dismatch	1.17 (0.69–1.96)	0.561	1.09 (0.60–1.97)	0.777
PNH, No vs. Yes	1.14 (0.46–2.86)	0.775	1.35 (0.46–3.93)	0.583
DSA, Negative vs. Positive	0.66 (0.31–1.41)	0.287	0.87 (0.37–2.05)	0.758
Infused CD34 + cell counts, ≤5 vs. >5	0.71 (0.41–1.21)	0.209	0.93 (0.51–1.71)	0.805
Conditioning regimen, FABT vs. Others	0.36 (0.16–0.79)	**0.011**	0.41 (0.18–0.97)	**0.044**
Letermovir, Yes vs. No	0.58 (0.29–1.18)	0.134	1.04 (0.45–2.41)	0.911
PLT Engraftment, Yes vs. No	0.23 (0.14–0.41)	**<0.001**	0.34 (0.17–0.71)	**0.004**
NE Engraftment, Yes vs. No	0.12 (0.06–0.28)	**<0.001**	0.04 (0.01–0.14)	0.071
CMV-Reactivation, Yes vs. No	1.81 (1.09–3.08)	**0.023**	1.19 (0.59–2.40)	0.620
EBV-Reactivation, Yes vs. No	0.87 (0.51–1.49)	0.603	0.98 (0.46–2.01)	0.964

Significant *p* values are in bold type.

## Discussion

The field of transplantation for AA has made remarkable strides in recent decades, with advancements in conditioning regimens significantly enhancing engraftment rates, reducing GVHD incidence, and improving OS. These improvements include strategies such as incorporating ATG, adjusting post-transplantation CTX dose (16), and combining immunosuppressive therapies (17). However, despite these achievements, conventional approaches appear to have reached a plateau. There is an urgent need for innovative approaches to further boost treatment efficacy, alleviate patient symptoms, and enhance quality of life.

GVHD remains a primary challenge in HID-HSCT for AA patients. The incidence of II-IV aGVHD varies between 12% and 42%, while cGVHD ranges from 20% to 56% (18–20), Current GVHD prevention focuses on immunosuppressive combinations. Though effective in reducing aGVHD, this approach increases the risks of organ dysfunction, infection, and relapse in cGVHD cases due to higher and prolonged immunosuppressive doses (21). MSCs are widely distributed in human tissues, with BM-MSCs being the first identified and containing the highest number of stem cells. They support HSCs homing and engrafting (22, 23), and possess low immunogenicity and strong immune regulatory functions (24). Previous studies indicate that MSCs can induce immune tolerance, alleviate aGVHD-induced inflammation, and have shown remarkable efficacy in treating steroid-resistant aGVHD (SR-aGVHD) (25). Notably, Le Blanc et al. (26) first used BM-MSCs to cure a child with SR-aGVHD, and Bonig et al. (27) achieved an OR rate of 80% in treating such patients. A phase II study confirmed that MSCs achieved a 70% OR rate and a 54% CR rate in SR-aGVHD patients, with good safety (28). Another phase III trial revealed that MSCs treated 54 SR-aGVHD pediatric patients, raising the OR rate to 70.4% on day 28 without infusion-related toxicity or safety issues (29). Also, Huang’s et al. (30) study indicated that early repeated MSCs infusions could lower cGVHD incidence and severity, and improve hemorrhagic cystitis symptoms. Zhang’s research (30) revealed that repeated infusions of MSCs at d+45 and d+100 effectively prevented cGVHD after HSCT, with only a 5.4% cGVHD incidence at 2 years. These studies have demonstrated the effectiveness of MSCs in treating SR-GVHD.

However, an increasing number of studies have revealed the preventive effect of early co-infusion of MSCs on GVHD. For instance, Wu et al. (31) treated 21 AA patients with Haplo-HSCT and MSCs co-infusion, resulting in II-IV aGVHD and cGVHD rates of 42.8% and 50%, respectively. In Liu’s et al. (5) study, the rates dropped significantly to 29.3% for II-IV aGVHD and 14.6% for cGVHD. Another study reported 28.4% for II-IV aGVHD and 26.8% for cGVHD (13). Chen’s et al. (32) research showed II-IV aGVHD at 20% and cGVHD at 19.3%. In our study, patients receiving early MSC co-infusion with HID-HSCT. Although no significant reduction in GVHD incidence was observed, there was a tendency for GVHD incidence to decrease in patients with MSC co-infusion compared to those without. Furthermore, MSC co-infusion significantly improved OS and GRFS, and consequently improved the quality of life of transplant patients.

Studies show that the mortality rate of HSCT can reach 22.3%, with 9.2% of infection-related deaths caused by viruses (33), predominantly CMV and EBV. AA patients often experience delayed immune reconstitution after HSCT, increasing the risk of CMV or EBV reactivation. Current treatments, such as broad-spectrum antiviral prophylaxis and preemptive therapy, have drawbacks like adverse effects and infection risks. In our study, viral reactivation rates were comparable between groups. However, the HID group saw four cases progress to EBV-related PTLD (*p* = 0.045). Several studies have indicated that although the use of letermovir can reduce the incidence of CMV reactivation, it may also contribute to the development of EBV-PTLD (34, 35). In our study, however, no significant difference was observed in letermovir usage between the two groups. Furthermore, the development of GVHD is closely associated with the occurrence of PTLD. Our data revealed a trend toward lower cumulative incidences of grade II-IV aGVHD and cGVHD in the HID+MSC group, although these differences did not reach statistical significance. This may partly explain the lower incidence of EBV-PTLD in this group. Therefore, we hypothesize that the immunomodulatory effects of MSCs may play a protective role in this context. Yet, MSC co-infusion showed a trend toward better treatment outcomes (OR rate: 82% vs. 90%), even if the difference wasn’t statistically significant. Because GRFS comprehensively reflects treatment efficacy and patient quality of life, we performed univariate and multivariate analyses of GRFS. In the univariate analysis, we found that the absence of MSC co-infusion, HCT-CI ≥ 1, CMV reactivation, poor NE and PLT engraftment, Bucy and FCA regimen were independent risk factors for GRFS. In the multivariate analysis, only PLT engraftment and the use of FABT regimen were associated with better GRFS (Table 3).

In conclusion, this study investigated the efficacy of HID-HSCT combined with MSCs co-infusion for SAA patients. The results indicated that MSCs co-infusion is associated with a reduction in cGVHD incidence without affecting engraftment or viral reactivation, thereby improving OS and GRFS. HID-HSCT combined with MSCs co-infusion represents a safe and effective therapeutic approach for patients with SAA. This single-center retrospective study is prone to recall bias. Also, factors like uniform conditioning regimen and GVHD-prophylaxis regimens need to be excluded. Future work should establish more standardized comparison protocols.

## Data Availability

The original contributions presented in the study are included in the article/supplementary material, further inquiries can be directed to the corresponding authors.
